# Supplementary material for: Resolution enhancement with deblurring by pixel reassignment (DPR)
Source: bioRxiv. 2023 Jul 24:2023.07.24.550382. Preprint. [Version 1] doi: 10.1101/2023.07.24.550382 (PMC10402078; doi:10.1101/2023.07.24.550382)
Supplement: Supplementary file 1 [file media-1.pdf]

Supplementary Material for: ”Resolution enhancement with deblurring by pixel reassignment (DPR)”

Bingying Zhao[a,*], **Jerome Mertz**[b]
[a]Department of Electrical and Computer Engineering, Boston University, MA 02215,
[b]Department of Biomedical Engineering, Boston University, MA 02215

* Corresponding author. byzhao@bu.edu

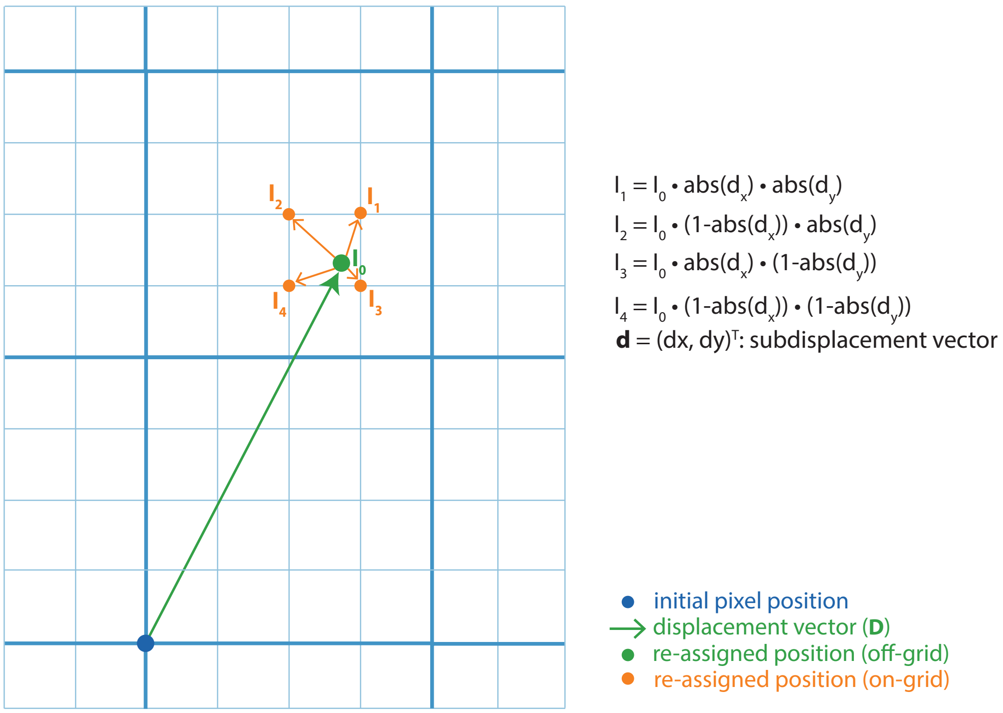

Fig S1 The working principle of weighted pixel reassignment.

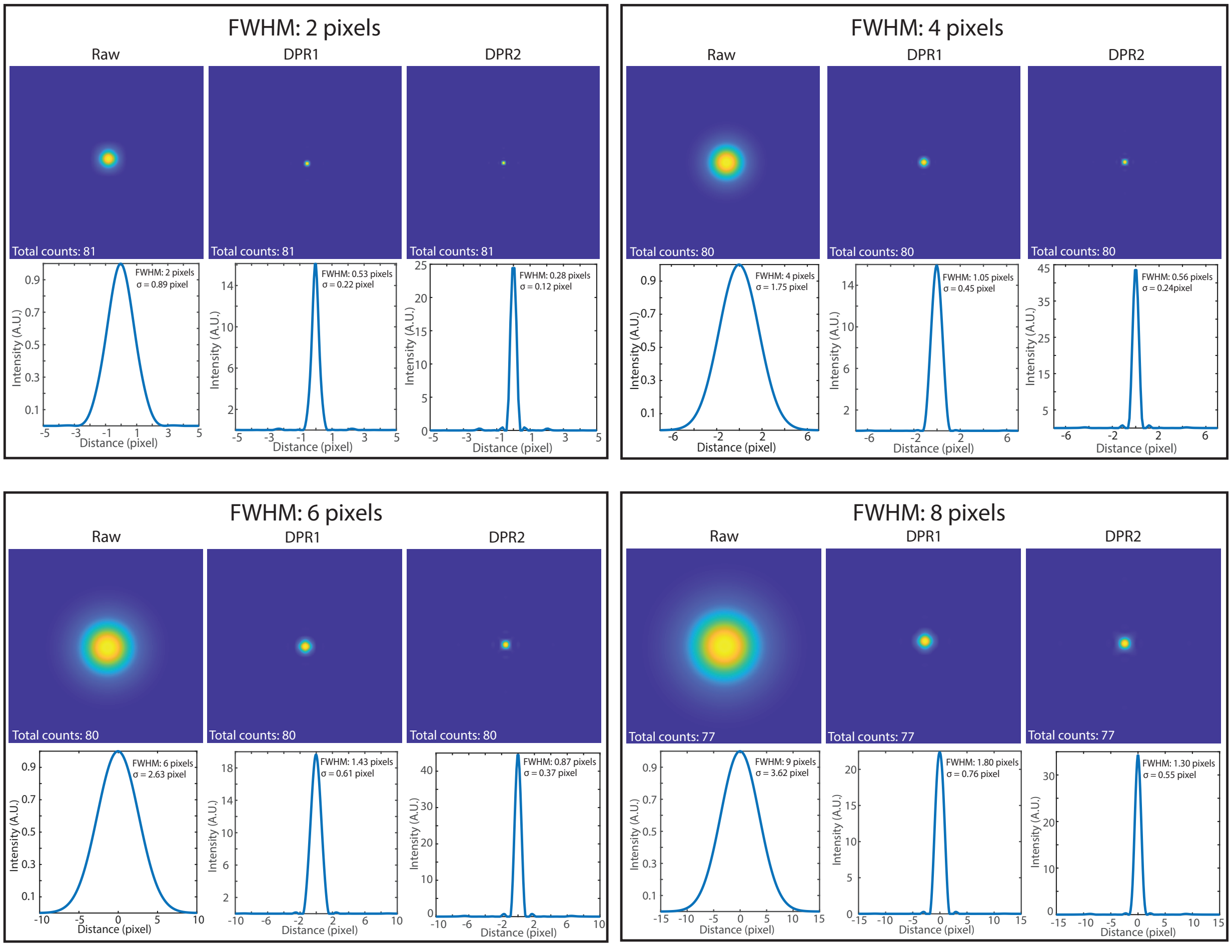

Fig S2 DPR applied to Gaussian PSFs of different sizes.

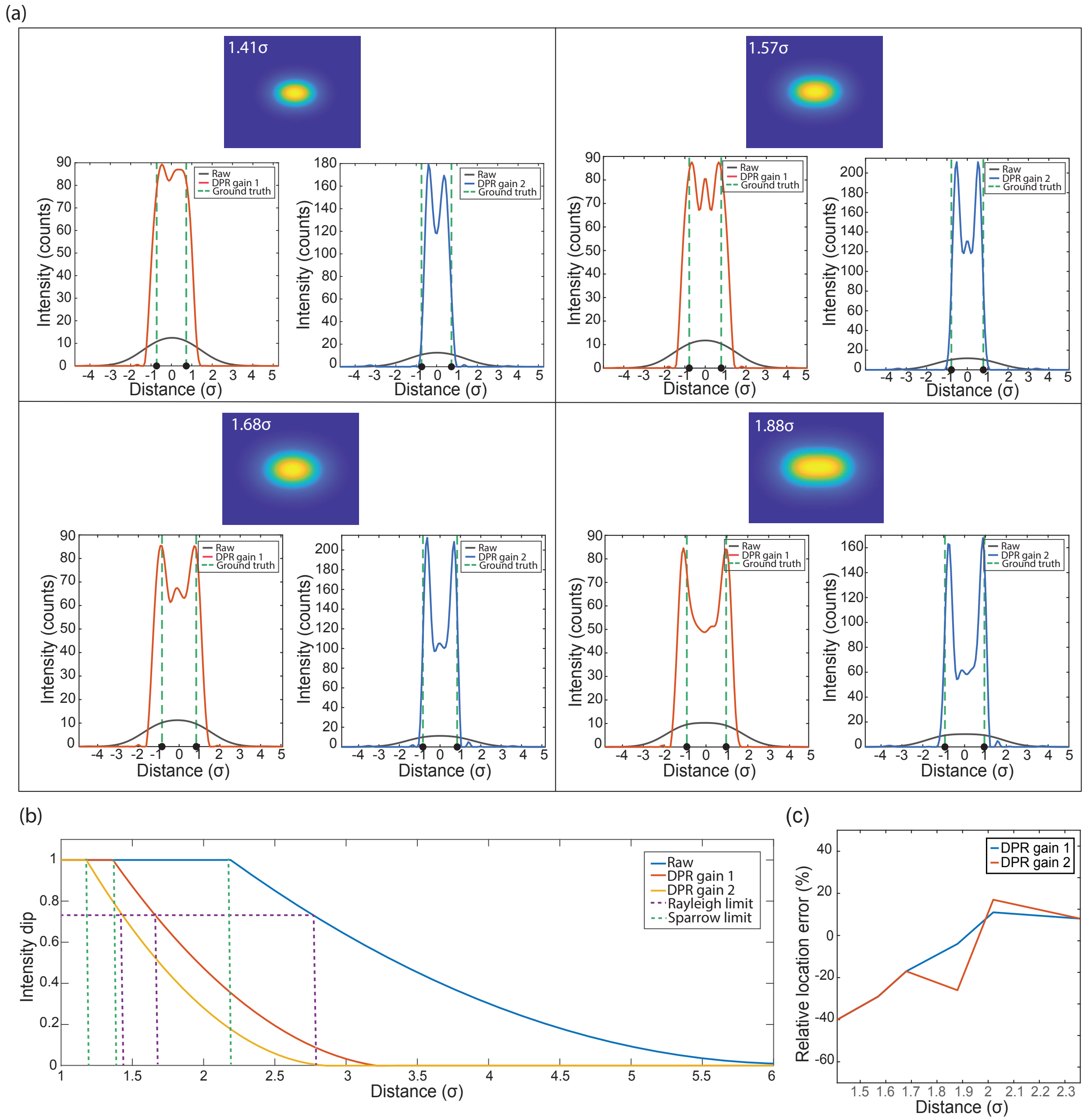

Fig S3 The separation accuracy of DPR. (a) DPR applied to simulated images of two point objects separated by different distances. (b) The resolution enhancements of DPR gain 1 and DPR gain 2. (c) The separation errors resulting from DPR.

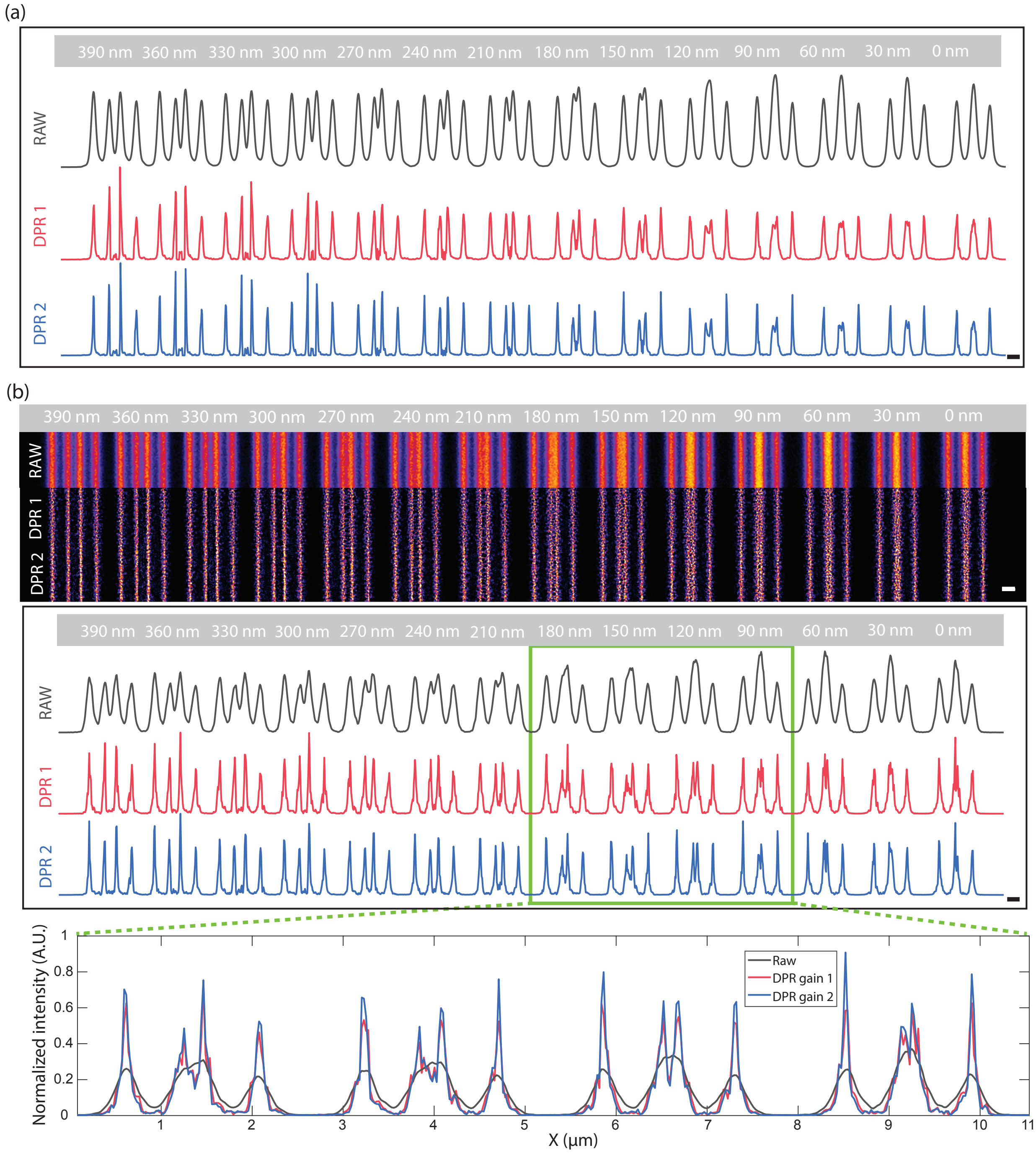

Fig S4 DPR applied to fluorescent lines of increasing separation from 0 nm to 390 nm. (a) Raw data acquired by an Airyscan microscope. The full set of line intensity profiles for raw, DPR gain 1, and DPR gain 2. Scale bar: 500 nm. PSF FWHM: 4 pixels. Sub window radius: 7 pixels. (b) Raw data acquired by a confocal microscope. Top: the full set of line images for raw, DPR gain 1, and DPR gain 2. Middle: the full set of line intensity profiles for raw, DPR gain 1, and DPR gain 2. Bottom: the expanded view of four line profiles for raw, DPR gain 1, and DPR gain 2, indicated by the green rectangle. PSF FWHM: 5 pixels. Sub window radius: 9 pixels. Scale bar: 500 nm.

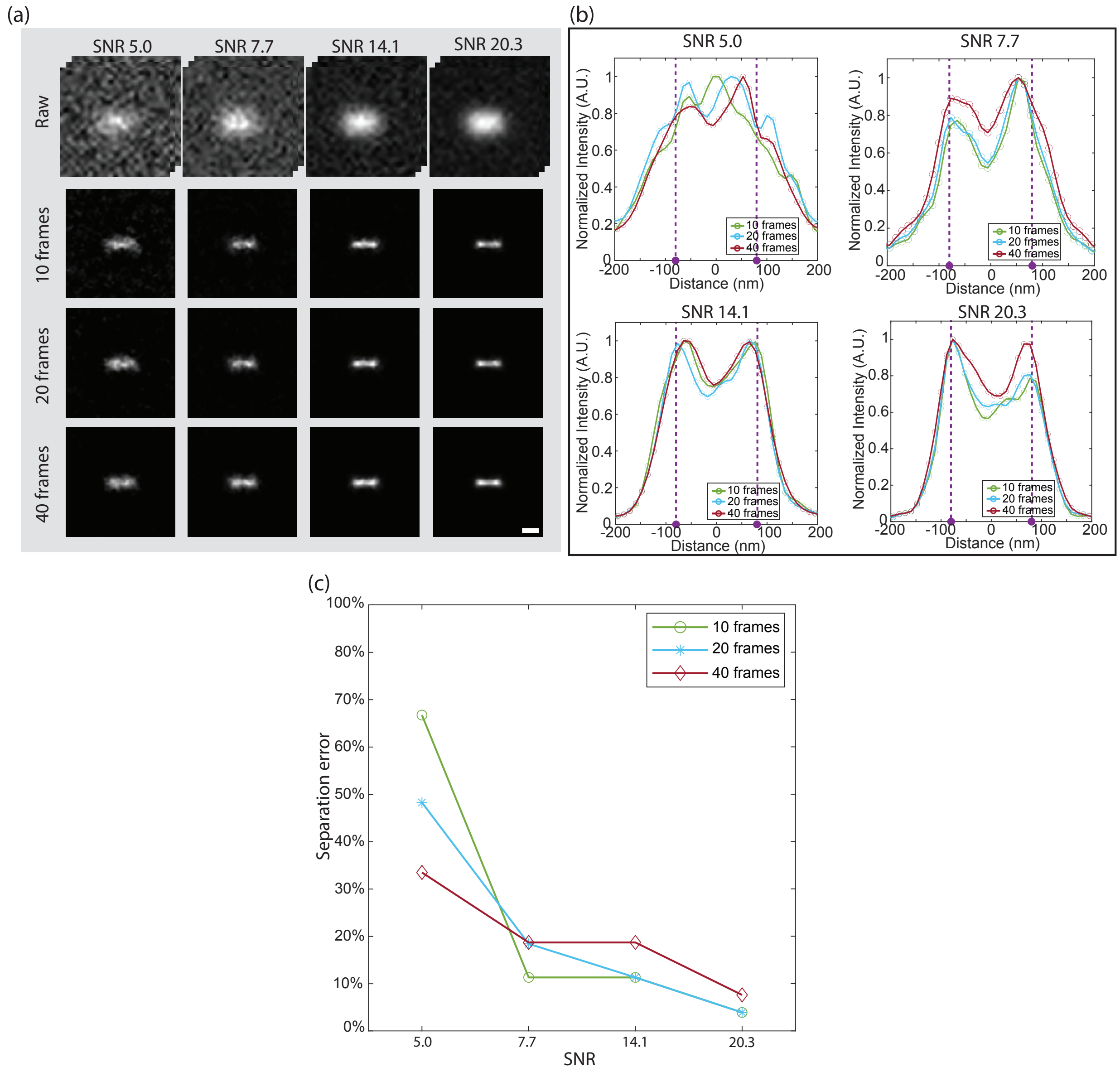

Fig S5 DPR applied to simulated images of different SNR. (a) Intensity images with different SNRs of the raw simulated two point objects separated by 160nm, without and with DPR. Scale bar: 100 nm. (b) Intensity profiles and (c) separation errors of the DPR reconstructed images when averaging over different numbers of frames. Dashed purple lines represent the ground-truth positions.

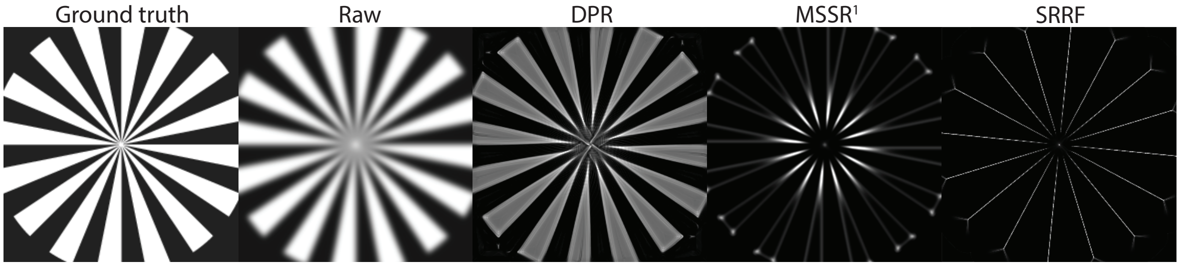

Fig S6 DPR, first order of MSSR, and SRRF applied to the simulated images of a Siemens star target.

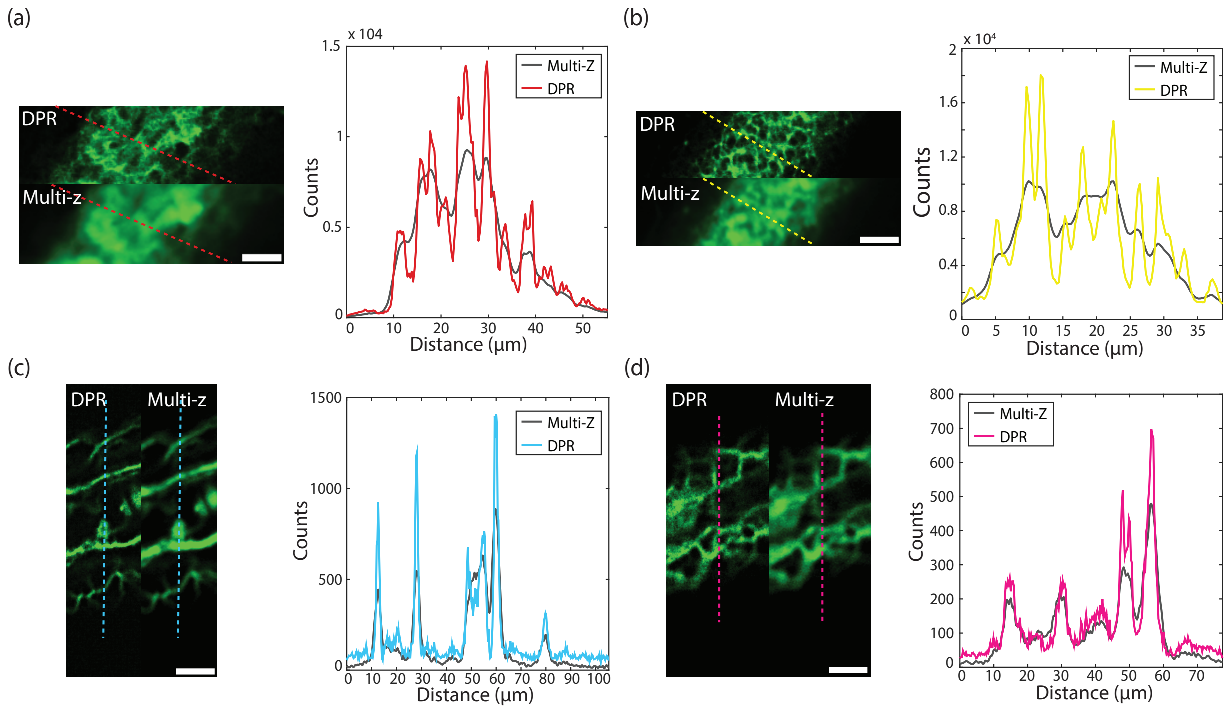

Fig S7 Zoomed-in ROIs of multi-z confocal zebrafish imaging. (a) ROI indicated by the red rectangle in Fig. 7(a), and intensity profile along the red dashed line. Scale bar: 10 μm. (b) ROI indicated by the yellow rectangle in Fig. 7(a), and intensity profile along the yellow. Scale bar: 12 μm. (c) ROI indicated by the cyan rectangle in Fig. 7(b), and intensity profile along the cyan dashed line. Scale bar: 18 μm. (d) ROI indicated by the magenta rectangle in Fig. 7(b), and intensity profile along the magenta dashed line. Scale bar: 13 μm

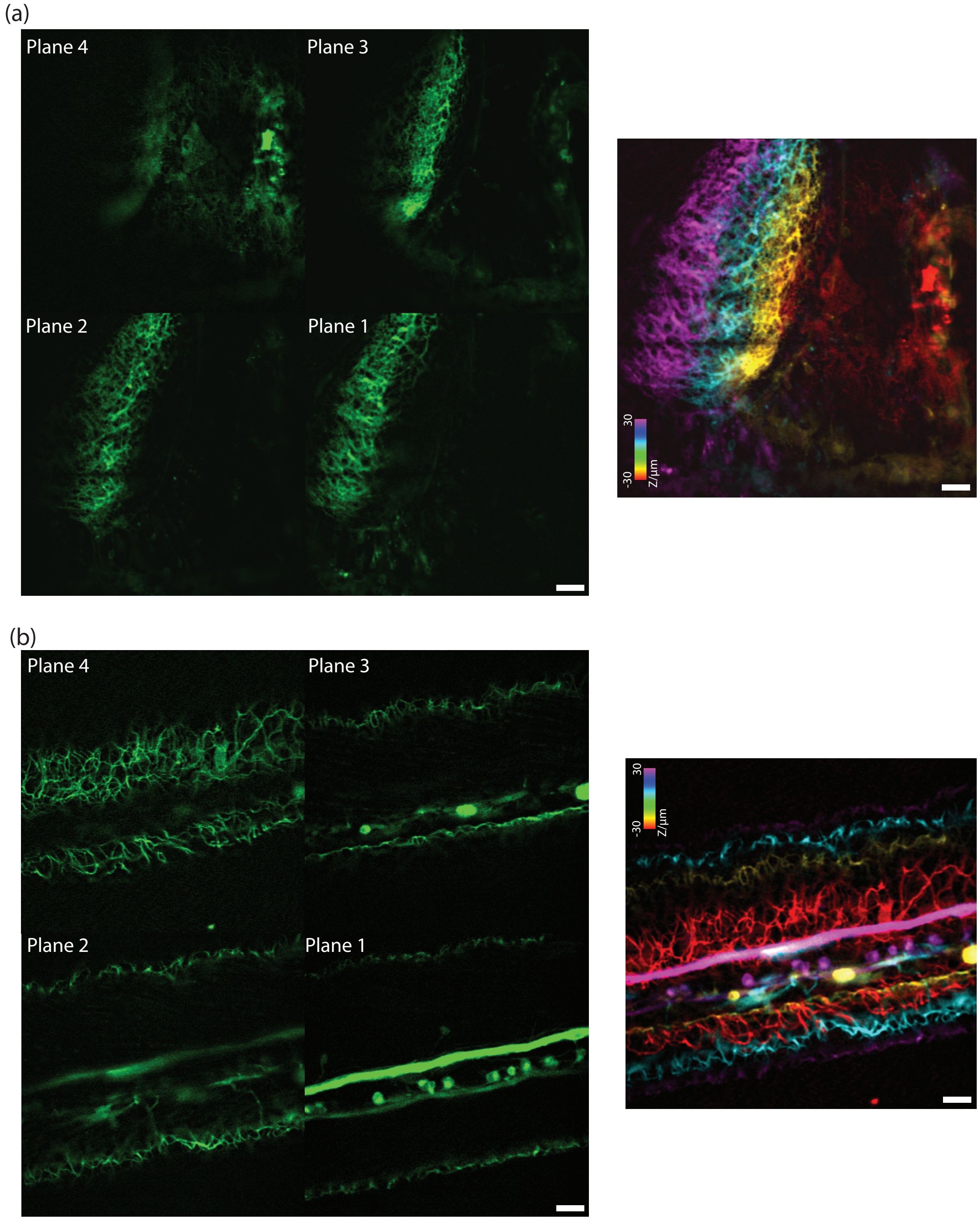

Fig S8 High-resolution multi-z confocal zebrafish imaging. (a) *In-vivo* multiplane images of the brain region in a zebrafish 6 dpf. Left: four image planes. Right: merged with color corresponding to depth. (b) In-vivo multiplane images of the zebrafish tail region in the same zebrafish. Left: four image planes. Right: merged with color corresponding to depth. Plane 1-4: the deepest to the shallowest. Inter-plane separation: 20 μm. Scale bar: 20 μm

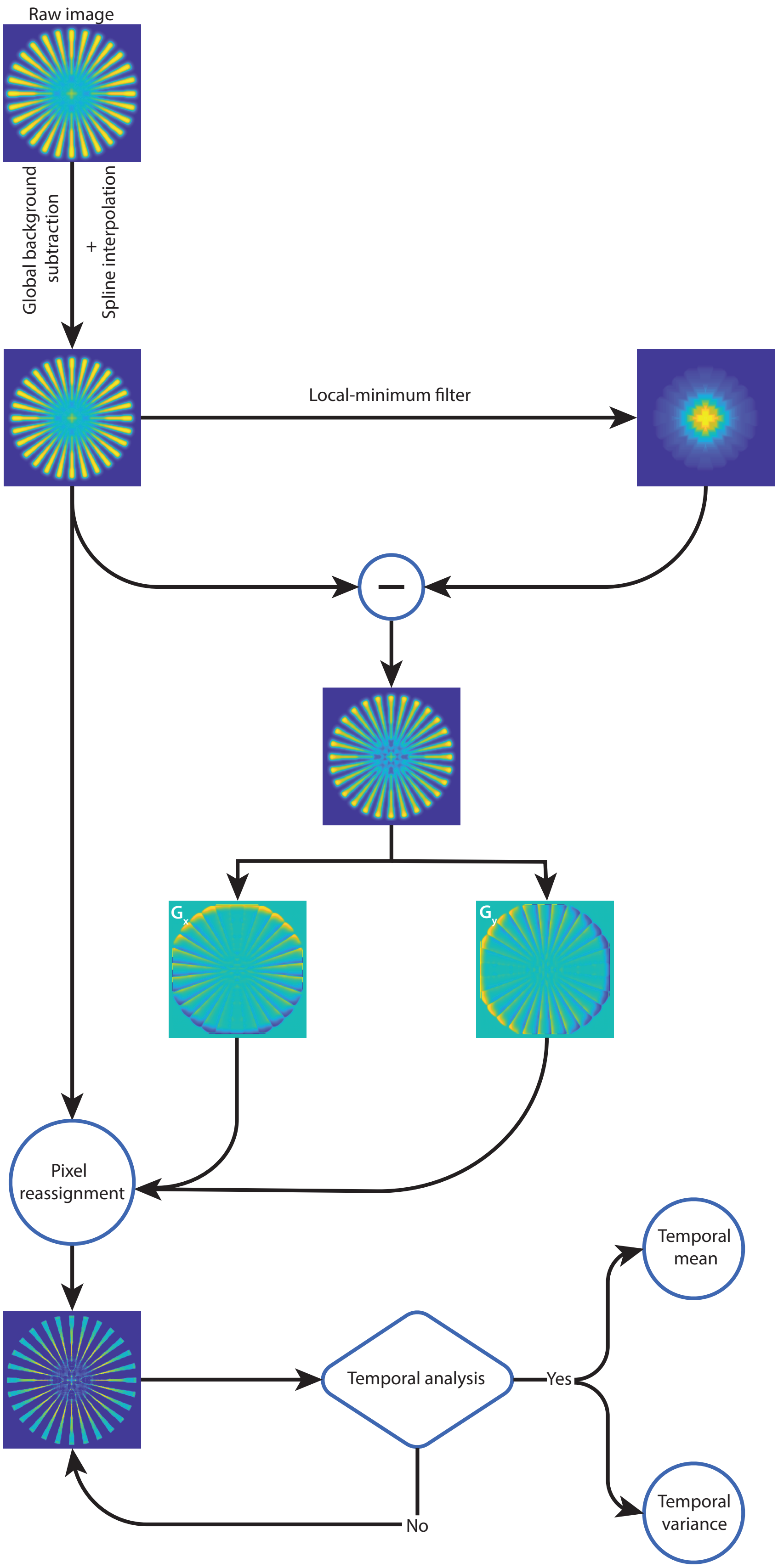

Fig S9 The overall workflow of DPR.

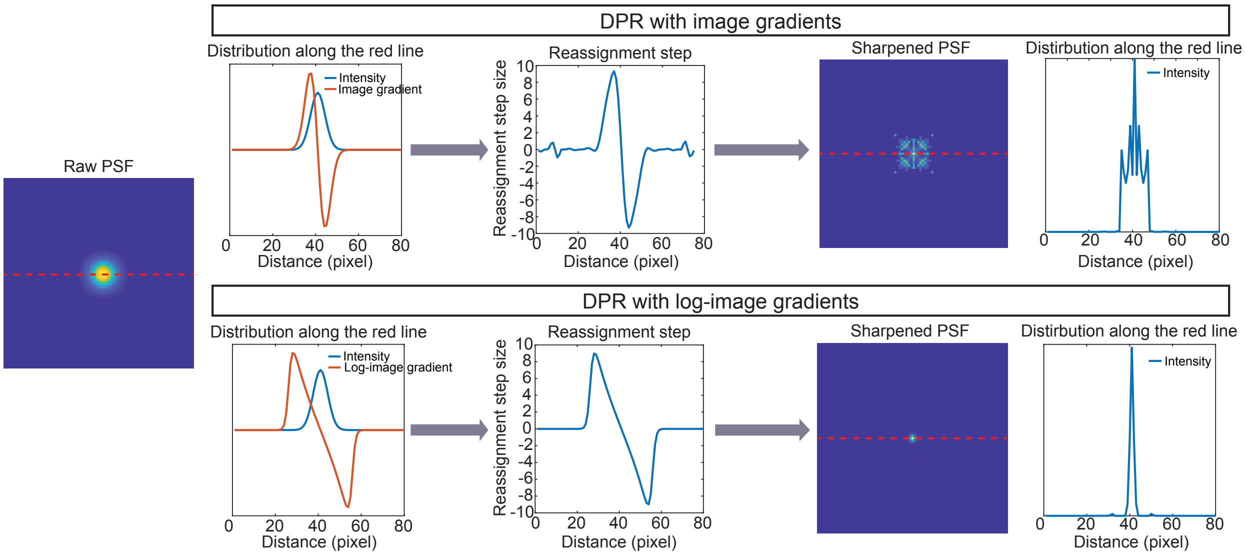

Fig S10 DPR using gradients compared to with log-PSF gradients.
